# Periodontal and Orthodontic Synergy in the Management of Stage IV Periodontitis: Challenges, Indications and Limits

**DOI:** 10.3390/life12122131

**Published:** 2022-12-16

**Authors:** Daniela Garbo, Mario Aimetti, Loretta Bongiovanni, Cristina Vidotto, Giulia Maria Mariani, Giacomo Baima, Federica Romano

**Affiliations:** 1 Private Practice, 10100 Turin, Italy; 2 C.I.R. Dental School, Department of Surgical Sciences, University of Turin, 10126 Turin, Italy

**Keywords:** malocclusion, orthodontics, pathologic tooth migration, periodontitis, periodontal treatment

## Abstract

This retrospective study described the clinical and radiographic long-term outcomes of combined periodontal and orthodontic treatment (OT) with fixed appliances in patients with Stage IV periodontitis and pathologic tooth migration (PTM) in the anterior sextants. OT was performed in either one or both arches, using tooth-supported or skeletal anchorage, following completion of active periodontal treatment and accurate planning of tooth movement biomechanics. Twenty-nine patients were identified and retrospectively examined when presenting for a supportive periodontal care (SPC) appointment. The mean SPC duration was 8.9 years (range 5 to 12 years). All anterior-migrated teeth showed statistically significant periodontal improvement compared to baseline values and stable radiographic bone levels at the final follow-up. Residual probing depths were 2.9 ± 0.5 mm at the end of active periodontal treatment, and they remained stable at the completion of OT (2.9 ± 0.6 mm) and at the last follow-up visit (2.8 ± 0.5 mm). These findings suggest that OT is a safe and effective treatment in improving the long-term prognosis of teeth with PTM in Stage IV periodontitis provided that periodontal health has been re-established and maintained with individualized SPC sessions.

## 1. Introduction

Periodontitis is the sixth most prevalent chronic disease in humans, affecting about 60% of the global adult population [1,2]. It is a bacteria-driven non-resolving inflammation that, if untreated, leads to tooth loss due to the progressive alveolar bone destruction with negative effects on chewing function and quality of life [2,3].

According to the 2018 classification of periodontal and peri-implant diseases and conditions, Stage IV periodontitis, in its most advanced form, is characterized by severe interproximal attachment loss, deep pocketing and bone resorption extending up to the root apex [4,5]. The reduction in periodontal support is generally associated with labial flaring, extrusion, spacing and drifting of the involved teeth, especially in the frontal area [6]. Maxillary incisors are particularly vulnerable to pathologic tooth migration (PTM) when posterior dental support has been lost [7]. These acquired occlusal changes, along with any underlying skeletal discrepancy, often result in a complex malocclusion and masticatory dysfunction that require complex multidisciplinary management [8]. The final goal is to restore function and aesthetics of the affected dentition and to improve the patient’s comfort and quality of life [8]. Such comprehensive treatment plans include steps 1 and 2 of periodontal therapy (elimination of individual risk factors, biofilm control and non-surgical periodontal treatment) followed by step 3 involving the surgical management of residual pocket sites, and subsequent orthodontic therapy (OT) [9].

The pivotal role of OT in the successful management of Stage IV periodontitis patients with PTM has been recently addressed in the European Federation of Periodontology guidelines [10]. The actual evidence supports the safe use of OT in patients with a periodontally reduced but healthy periodontium provided that the results of steps 1–3 of periodontal therapy are maintained during tooth movements [11,12]. Moreover, OT would seem to have some beneficial effects on periodontal parameters of pathologically displaced teeth [13]. Indeed, proper control of periodontal infection together with adequate OT and prosthetic rehabilitation, when needed, represents an effective strategy to improve the prognosis of the entire dentition [10].

Orthodontic correction of PTM is a demanding procedure requiring specialized skills to plan the movement, anchorage, and biomechanics according to the loss of periodontal support [8]. As in most cases, there is a need of intrusion and retraction to correct flaring, over eruption and embrasures in the frontal area. Extrusion is often indicated to correct the uneven gingival margins in case of excessive gingival display and high-aesthetic need, as vertical tooth movements can effectively level the soft tissue position and improve the smile appearance [14]. In this case, there is a need for additional endodontic and restorative treatments of the extruded teeth. Mesio-distal movement and rotations may be performed in cases in which space distribution, black triangles or crowding must be corrected. When planning any tooth movement, it is mandatory to assess the available bone support, root anatomy, positioning of the centre of resistance (CR) as well as the magnitude and direction of the force system necessary to achieve the planned final tooth position [15]. Because of periodontal bone loss, CR is apically displaced on the involved teeth, making the biomechanics of bodily tooth movements more complex.

Results from an animal study by Kondo et al. indicated that the type of orthodontic tooth movement, bodily or tipping, did not influence the displacement of CR, but the rate of cervical bone resorption, which was enhanced by the latter [16]. Thus, it is recommended to apply bodily movements on teeth with reduced periodontal tissue support. Indeed, mistakes in the planning of OT may cause detrimental effects that can jeopardize the prognosis of the involved teeth, with root resorption and bone dehiscence formation being the most common [17,18]. Furthermore, the introduction of fixed orthodontic appliances into the oral cavity also favours the accumulation of dental biofilms, thus increasing the risk of recurrence of inflammation [19].

At present, the interaction between OT and periodontal treatment has been poorly documented in the long-term [11,12,20,21]. As the number of adults suffering from Stage IV periodontitis is increasing [22] and many of them are seeking OT to correct PTM and to retain their teeth [23], this question is clinically relevant. Therefore, the aim of the present study was to describe the long-term outcomes of a multidisciplinary treatment of Stage IV periodontitis patients and to address the challenges, limits and indications of OT in such cases.

## 2. Materials and Methods

### 2.1. Study Design and Population

This was a retrospective study on the combined effect of periodontal and orthodontic treatment in Stage IV periodontitis patients. The Institutional Ethical Committee approved the study (n° 2028/2016) and all participants gave informed consent before enrolment.

Consecutive subjects suitable for the study were recruited during supporting periodontal care visits (SPC) between February and July 2022 from the population treated for severe periodontitis and PTM in a private periodontal office in Turin, Italy. The following inclusion criteria were considered: (1) age ≥ 18 years at the first visit; (2) good general health with women not pregnant or lactating; (3) no heavy smoking; (4) presence of generalized Stage IV periodontitis (severe generalized chronic periodontitis according to the previous classification) with at least ≥4 sites around anterior teeth with clinical attachment level (CAL) and probing depth (PD) ≥ 8 mm; (5) PTM of upper and/or lower anterior teeth treated using fixed orthodontic appliances; (6) complete clinical and radiographic documentation at baseline (T0), at the completion of active periodontal treatment (T1), at the end of OT (T2) and at the last SPC session (T3); and (7) compliance with SPC (≥3 visits per year).

### 2.2. Active Periodontal Treatment

**Figure 1:**
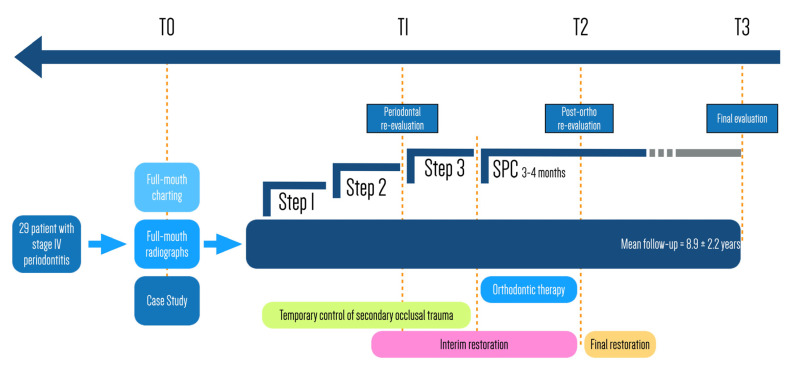
Flow chart of the study and comprehensive multidisciplinary treatment sequency.

Figure 1 provides a flow chart of the study design and the comprehensive treatment plan. Following the baseline examination (T0), including full medical and dental history, full-mouth periodontal charting and radiographic analysis, all patients received active periodontal treatment. This consisted of oral hygiene motivation and instructions, risk factor control, conventional cycle of non-surgical therapy, extraction of hopeless teeth and additional resective or regenerative periodontal surgery when needed. Experienced dental hygienists carried out the non-surgical treatment, while the same expert periodontist (M.A.) performed the surgical interventions.

### 2.3. Orthodontic Therapy

OT started 3 to 6 months following the completion of active periodontal therapy (T1) if the resolution of inflammation (full-mouth bleeding score (FMBS) < 20%) and the absence of pathological sites (PD > 4 mm) were attained and patients could display an adequate home control of dental biofilm (full-mouth plaque score (FMPS) < 20%). The goals were to stabilize occlusion, to achieve correct overbite and overjet, to restore proper anterior guidance, to solve crowding or spacing, and to improve aesthetics.

An experienced orthodontist (D.G.) performed OT using the principles of the segmented arch technique with light continuous forces [24]. Full-fixed edgewise-orthodontic appliances with bonded brackets were used either in the upper or lower arch or in both arches. Brackets were either stainless-steel or ceramic with Roth prescription, slot size 0.022 × 0.028 inches. Molar stainless-steel bands or buccal-bound stainless-steel tubes were used, both with double rectangular tubes for possible insertion of auxiliary arch wires.

The tooth movement was accurately planned before starting the treatment with precise calculation of force direction, magnitude and point of application to avoid undesirable tooth movements. Initial levelling was performed with the insertion of 0.014 inches copper–nickel–titanium arch wires. After the first phase of levelling, the posterior anchorage segments were secured with passive 0.018 × 0.025 stainless-steel wires. The same passive wire was inserted in the anterior segment to be intruded/lingually tipped. The active force for intrusion and the retraction-space closure were obtained by inserting one cantilever in a titanium–molybdenum alloy (TMA) on each side of the auxiliary molar tube. The size of the TMA wire was 0.017 × 0.025 inches to allow low and continuous force for optimal tooth movement. Logarithmic shape cantilevers were used to obtain simultaneous intrusion and retraction [25]. A three-piece base arch was used for simultaneous intrusion and retraction according to Burstone and Shroff [26].

The applied force was approximately 10 g per tooth, keeping it as light as possible for optimal tooth movement and bone remodelling. Reactivation was performed every 8–10 weeks, with the patients being checked every 4 weeks.

Elastic power chains were occasionally used according to the planned tooth movement to achieve the final tooth position. Posterior anchorage was reinforced occasionally with the aid of temporary anchoring devices or dental implants as they provide ideal anchorage units.

During OT, patients were kept on monthly recalls by a dental hygienist. At the end of OT, they received a fixed-bound retention on the lingual tooth surface and a vacuum-formed removable retainer for night time use to support occlusion and to avoid tooth relapse in case of accidental debonding of the fixed retainer. Patients were advised to wear the retainer for life.

### 2.4. Supportive Periodontal Care

At the completion of the combined periodontal and orthodontic treatment (T2), patients received definitive prosthetic rehabilitation and were enrolled in the SPC with individualized intervals of 3–4 months. At each recall they were reinforced in home oral hygiene measures and received professional tooth cleaning by experienced dental hygienists. Full-mouth periodontal parameters were recorded yearly during the SPC.

### 2.5. Smile Aesthetic Evaluation

Smile aesthetics were scored by all the enrolled patients and by 10 expert clinicians based on photographs taken at T0 and T2. They were asked to rank their satisfaction with aesthetics of the anterior teeth considering four elements (tooth appearance, tooth shape, tooth alignment, and gingival display/position of the gingival margin) using a five-point Likert scale (1 = not satisfied, 2 = moderately dissatisfied, 3 = neither satisfied neither dissatisfied; 4 = moderately satisfied; 5 = completely satisfied).

### 2.6. Clinical Periodontal Parameters

After pseudonymization, clinical data measured at T0, T1 and T2 and at the time of the latest SPT visit (T3) by a single examiner (M.A.) were entered into a data set for statistical analysis. The clinical parameters included presence/absence of plaque (PI), presence/absence of BoP, PD, gingival recession (REC) and CAL at six sites per tooth except for third molars. FMPS and FMBS were recorded as the percentage of total surfaces with PI and BoP, respectively. Reasons for tooth extraction (endodontic failure, caries, vertical fracture, periodontal or unknown reasons) were also assessed.

### 2.7. Radiographic Periodontal Parameters

In each patient, periapical X-rays of maxillary and mandibular anterior teeth were taken at T0, T1 and T3 using the parallel technique. The radiographs were digitized and imported into the ImageJ software (NIH, Bethesda, MD, USA) to measure the marginal bone levels (MBL) and root length (CEJRA) using an electronic ruler at a 10× magnification. A reference line was drawn joining the mesial and distal aspects of the cement–enamel junction (CEJ) and the perpendicular distance from this landmark to the marginal bone was identified as the MBL [27]. The CEJRA was measured as the distance between the CEJ line and the tooth apex. Then, the percentage of interdental residual bone was calculated with respect to the CEJRA on the mesial and distal surfaces. If the CEJ (or margin of restoration) or the alveolar crest could not be identified in any of the consecutive radiographs, the site was excluded. The percentage of residual bone and the CEJRA were assessed twice for all sets of radiographs by a calibrated examiner (C.V.), and the mean of the measurements was used in the analysis.

### 2.8. Data Analysis

The primary outcome variable was CAL change on anterior teeth with PTM. Quantitative data were summarized as means and standard deviation (SD) and categorical data as absolute and relative frequency distributions. Full-mouth data and data from migrated anterior teeth were separately analysed. Statistical significance of the changes over time in clinical and radiographic parameters was verified using the repeated measures of analysis of variance (FMBS, CAL, radiographic measurements) and the Friedman test (FMPS, PD, REC), followed by post hoc tests for multiple comparisons (Tukey’s test and Dunn’s test). Fisher exact test was used to compare the degree of satisfaction with smile aesthetics between patients and clinicians. A *p* value < 0.05 was considered statistically significant. Statistical analysis was performed using SPSS software (version 28, IBM, Armonk, NY, USA).

## 3. Results

### 3.1. Patients

The records of 29 subjects (23 females, 6 males) who met the inclusion criteria were reviewed. The mean age was 55.1 ± 6.5 years (44 to 68 years); four patients were light smokers (≤6 daily cigarettes). Patients were treated between January 2006 and December 2016 and were maintained for 8.9 ± 2.2 years (5 to 12 years).

### 3.2. Orthodontic and Aesthetic Treatment Outcomes

All patients had Class I or Class II malocclusion, increased overjet and overbite up to 10 mm and 9 mm, respectively. Extrusion and migration of the anterior teeth were observed together with spacing or crowding of lower incisors and lip catching.

The mean OT time was 19.9 months, ranging from 4 to 38 months; 12 patients received fixed OT at upper and lower anterior sextants, 16 subjects only at the upper arch and only one at the lower arch. At the end of OT, correct overjet and overbite ranging from 2 to 4 mm were achieved, together with closed contact points, elimination of occlusal trauma and lip catching, midline alignment and correct spaces for prosthetic restorations. Interincisal angle was normalized to approximately 132–135° to provide good anterior support.

**Figure 2:**
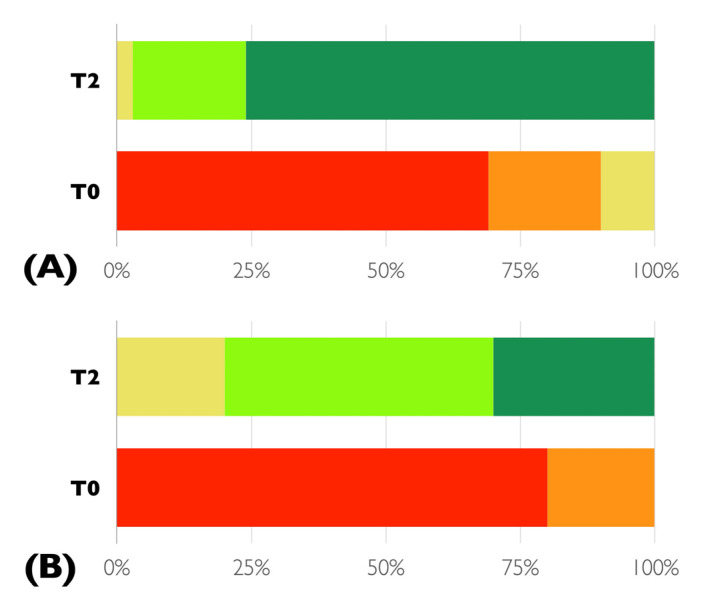
Frequency distribution of the degree of satisfaction of smile aesthetics by patients (**A**) and clinicians (**B**) before (T0) and after the completion of the combined periodontal–orthodontic treatment (T2) according to the five-point Likert-scale. Red = not satisfied, orange = moderately dissatisfied, yellow = neither satisfied neither dissatisfied, light green = moderately satisfied, dark green = completely satisfied.

As shown in Figure 2, the majority of the patients were dissatisfied with their own smile at T0, while at the completion of the combined periodontal–orthodontic treatment most of them were completely satisfied. There was a statistically significant difference in the perception of smile aesthetics by patients and clinicians at T2, with patients scoring higher than clinicians did (*p* = 0.026).

### 3.3. Periodontal Clinical and Radiographic Outcomes

Overall clinical measurements at baseline, T1, T2 and T3 examinations are summarized in Table 1. FMPS and FMBS decreased significantly at T1 compared to baseline values and remained below 20% during the observational period. After active periodontal treatment CAL and PD significantly improved from baseline (*p* < 0.001). A mean CAL gain of 0.6 ± 0.9 mm was achieved together with a significant PD reduction (1.1 ± 0.8 mm). At T2 and T3 CAL and PD did not significantly change from T1, remaining significantly improved with respect to the baseline values.

Changes in clinical and radiographic periodontal variables at migrated anterior teeth are shown in Table 2. At T1 anterior teeth experienced a mean CAL gain of 0.8 ± 0.9 mm and a mean PD reduction of 1.4 ± 0.9 mm (*p* < 0.001). Clinical parameters remained stable at T2 (*p* > 0.05) and during the course of SPC (T2–T3). During the observational period the alveolar bone support levels were unchanged, whereas CEJRA decreased on average from 16.3 ± 1.6 mm at T0 to 14.7 ± 1.8 mm at T3 (*p* < 0.01).

As reported in Table 3, during the observational period a total of 82 hopeless teeth were extracted, mostly for periodontal reason (67%). Seventy-two teeth were extracted during the active phase of the periodontal treatment (T0–T1), four molars used for orthodontic anchorage were extracted at the completion of OT (T1–T2) and six premolars were lost during SPC (T2–T3) to root fracture. Lost teeth were replaced with tooth- or implant-supported fixed restorations, as indicated.

**Figure 3:**
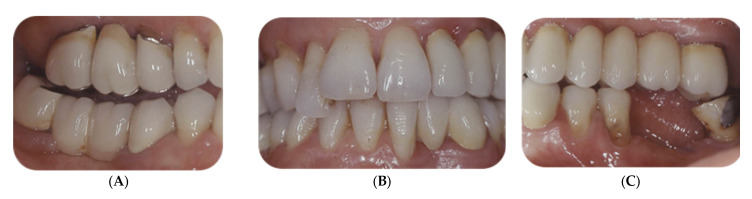

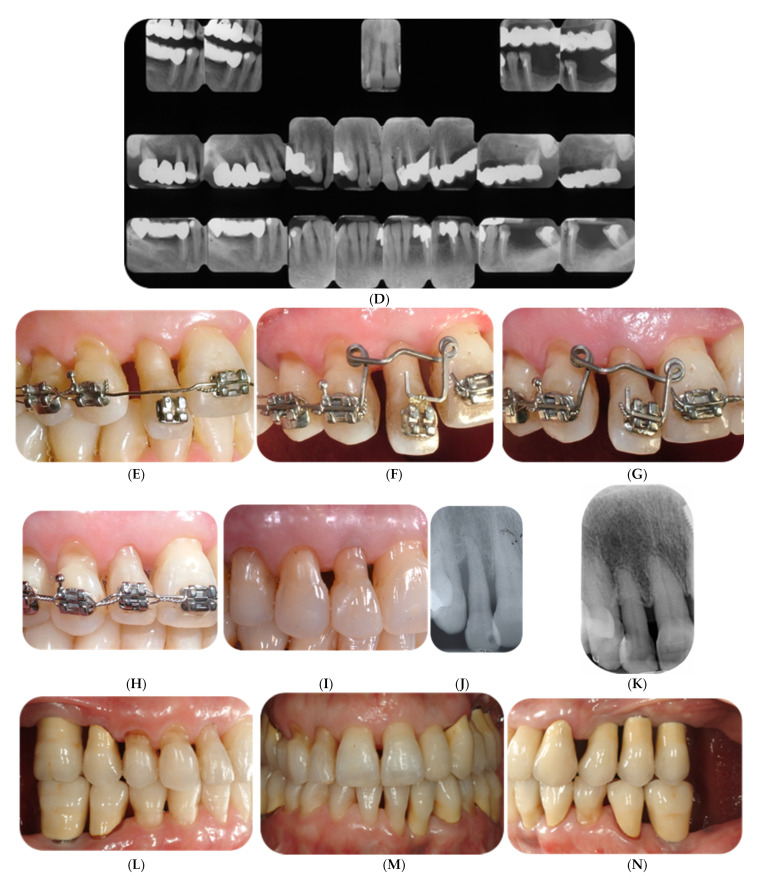

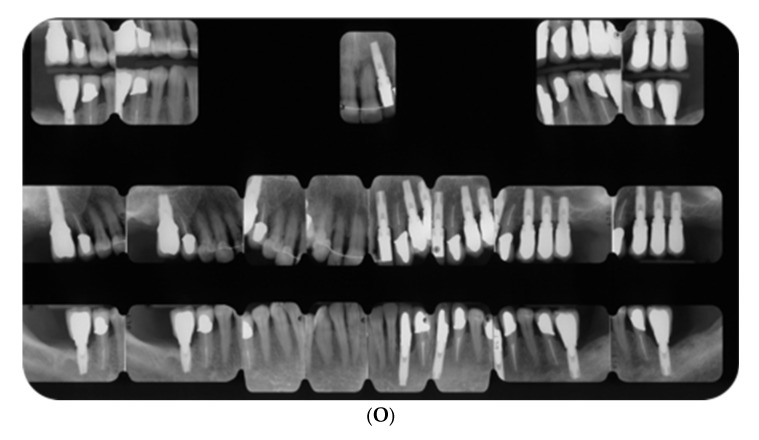
A fifty-two-year-old female with generalized Stage IV periodontitis, PTM of tooth 12 in occlusal trauma. (**A**–**C**): Baseline clinical view. (**D**): Baseline full-mouth radiographs. (**E**–**I**): Fixed OT following active periodontal therapy. First, minor levelling and space opening for proper alignment of tooth 12 were obtained. Thereafter, tooth 12 was intruded according to the segmented arch technique, using an implant anchorage on tooth 16 and a cantilever activated with light forces of 10 g. Treatment lasted 10 months, thereafter the patient was referred for final prosthetic rehabilitation. (**J**,**K**): Periapical radiographs of tooth 12 at baseline and at T3. (**L**–**O**): Clinical view and full-mouth radiographs after 12 years of SPC. Implant-supported restorations were placed to replace missing or extracted teeth during the initial phase of periodontal treatment. No additional teeth were lost.

An exemplificative case of combined periodontal–orthodontic treatment and long-term outcomes is described in Figure 3.

## 4. Discussion

OT is nowadays considered a fundamental part of the multidisciplinary treatment of patients affected by Stage IV periodontitis [10]. The present findings support the safety and the efficacy of OT in improving the long-term prognosis of teeth with PTM provided that periodontal health has been re-established and maintained with individualized SPC sessions.

Due to the reduced periodontal support, Stage IV periodontitis patients develop a secondary malocclusion caused by forces acting in the oral cavity such as tongue pressure, chewing forces, and lip impingement [6]. Posterior teeth without an antagonist in the opposite arch tend to extrude, while frontal teeth tend to both extrude and move buccally as they do not have anterior contacts counteracting these forces [6,7]. Patients included in the current study presented either a class I molar relationship with increased overjet and overbite and diastemas on the frontal teeth, or a class II molar relationship due to pre-existing malocclusion with migrated anterior teeth and increased overbite. Crowding or spacing was also present according to the initial malocclusion. An anterior crossbite of single teeth, such as lateral incisors, was detected in some patients, due to buccal migration of lower incisors and lingual migration of upper lateral incisors. Concomitantly, occlusal trauma of anterior teeth was found, due to extrusion of anterior teeth combined with the lack of posterior tooth support. Some patients showed mesio-distal migration of canines, premolars, or molars due to missing neighbouring teeth, causing poor occlusion and difficulties in the prosthetic rehabilitation.

These patients require a complex interdisciplinary treatment that should be carefully planned based on their full-mouth records (periodontal charting, dental models, intraoral and extraoral photos, lateral cephalograms and periapical radiographs) and reassessed at the completion of the active periodontal treatment [8]. Patient problems have to be listed and possible solutions discussed thoroughly with all professionals and presented to the patient for the final decision. This phase allows healthcare team members to schedule the steps and timing of the different interventions for an integrated work flow [28]. The initial treatment stage always involves dental, non-surgical and surgical, if needed, periodontal therapies, followed by OT and finally by prosthetic restoration. It is imperative to perform orthodontic tooth movements in reduced but healthy periodontium (PD ≤ 4 mm without BoP) as movements of teeth within inflamed periodontal tissues were found to increase the loss of periodontal attachment [10,13,29,30]. Regarding timing for initiating OT after periodontal surgery there are no established guidelines [21]. Mathews and Kokich proposed to start tooth movements 3 to 6 months after periodontal surgery [31]. Pini Prato and Chambrone suggested postponing OT after the complete healing dynamics of the periodontal tissues, which required up to 12 months in case of periodontal regeneration [32]. We started OT 3 to 6 months following active periodontal treatment, provided that patients could demonstrate adequate biofilm control.

At the time of bracket placement, it is essential to instruct patients in modifying their home oral hygiene manoeuvres and to schedule strict periodontal maintenance sessions in order to monitor their compliance throughout OT [33]. Effective plaque removal is more difficult to accomplish in the presence of fixed orthodontic appliances [17]. Orthodontic retention and SPC are the last to be planned to stabilize the treatment outcomes in the long-term.

In adult patients with periodontitis the goals of OT may differ from those in patients with healthy periodontium, as tooth movements show some limitations [11,34]. While it is not always feasible to reach a perfect class I molar relationship, it is essential to obtain a correct overbite and overjet, close contacts between teeth, and a posterior occlusal support with balanced contacts.

In the present study, tooth movements were carefully planned to adapt the biomechanics to the individual anatomic features, as the reduction in periodontal support causes the apical migration of the tooth’s CR. In this situation, when applying forces at the bracket level, uncontrolled tipping may occur more easily, while controlled tipping, bodily and root movements are more difficult to obtain as the line of action needs to be planned more apically. Therefore, careful biomechanics planning is needed in order to avoid undesired effects leading to further attachment loss.

In the frontal region the treatment goals may differ according to the gingival display at smile. In fact, both extrusion and intrusion are described as possible tooth movements to solve secondary malocclusion in periodontitis patients. Patients with gum display have higher aesthetics expectations, while patients who do not show the gingival margin need primarily crown levelling and are more prone to accept a compromise on gingival levels. Indeed, intrusion is the movement of choice to level extruded teeth and to preserve the natural dentition, while extrusion, endodontic treatment and final prosthetic restoration are preferred in the case of gum display, as extrusive movement can level hard and soft tissues helping to restore an aesthetic smile [14].

Conversely, indication for intrusion is the presence of over-erupted teeth with no pathological PDs, when the treatment goals include correction of excessive overbite, tooth alignment and levelling and maintenance of natural teeth, thereby minimizing the use of prosthetic restorations in patients with a low smile line. In the segmented arch technique, intrusion is performed applying a single constant force of about 10 N cm in the apical direction along the long axis of the tooth, minimizing apical root resorption [35]. Cantilevers or intrusion wires made of 0.017 × 0.025 inches TMA are tied by a metal ligature with one point contact to the teeth to be intruded. If more than one tooth requires intrusion, a passive stiff segment made of 0.018 × 0.025 stainless-steel wire is inserted on all the anterior teeth. These mechanics, called “statically determined systems”, enable to calculate forces and moments delivered to the teeth in the active and reactive units. As an alternative, intrusion can be obtained with statically undetermined systems, but tooth movement is less predictable. After intrusion, improvement of clinical crown length and marginal bone level may occur, while it still remains questionable if a new attachment can be attained [35,36,37,38].

The choice of the anchorage is fundamental, as most of the patients with stage IV periodontitis have experienced the lack of posterior teeth or have teeth offering poor anchorage due to reduced periodontal support. The possibility of using prosthetic implants, temporary anchorage devices (TADS) such as miniscrews, or natural teeth should be taken into consideration. Hopeless teeth can be retained during OT to maintain the occlusal support and used for anchorage, thus postponing their extraction to a later phase of treatment [31].

Finally, the selection of the orthodontic appliance to be used is strictly dependent on its capability of developing the forces required to obtain the planned tooth position. Fixed appliances seem to be more effective compared to clear aligners [39].

Regarding the effect of fixed OT on periodontal tissues, the majority of data in the literature are short-term and most of them refer to the orthodontic movement of teeth with infrabony defects [21,40]. There is a need for studies with longer follow-up periods. In the current study full-mouth mean CAL gain and PD change achieved with active periodontal treatment (0.6 ± 0.9 mm and 1.1 ± 0.8 mm) were maintained following OT (0.8 ± 1.1 mm and 1.3 ± 0.9 mm) and during the 8.9 years of SPC (0.6 ± 1.0 mm and 1.2 ± 0.8 mm) with all anterior teeth with PTM being successfully retained. This enforces the beneficial effect of orthodontic correction of PTM for periodontal stability [8]. The present data are in line with the clinical improvements reported in previous research [12,41,42]. Moreover, they are supported by the evidence arising from a recent systematic review on the absence of a clinically relevant effect of OT on CAL and PD changes in patients with reduced but healthy periodontium after combined treatment [11]. Conversely, other studies reported greater PD and CAL changes after combined periodontal–orthodontic treatment than those obtained in the current study [11,13]. It is worth noting that the present data were full-mouth, while findings from other studies refer only to pathological sites.

Radiographically, we obtained a mean apical root resorption of 1.6 mm at T3 that was partially compensated for by a progressive increase in alveolar bone during SPC. It could be hypothesized that some degree of bone remineralization occurred and that intrusive orthodontic movements promoted new attachment on reduced but healthy periodontium [38]. Consistently, the systematic review by Papageorgiou et al. reported an improvement in MBL of 0.36 mm after the combined treatment of pathologically migrated teeth [13]. Previous studies by Melsen [35,43] and by Artun and Urbye [44] demonstrated little or no marginal bone loss around orthodontically intruded teeth provided that inflammation was under control.

Few and inconsistent data are available in the literature on root resorption following orthodontic intrusion, with Corrente et al. [36] observing no resorption and Melsen et al. [35] describing 1–3 mm of root resorption. However, radiographic measurements should be considered only suggestive for bone and tooth modifications due to the changes in tooth inclination following OT.

Patient’s expectation, and level of satisfaction are important parameters to measure the overall treatment outcome. Nonetheless, data on satisfaction among adult patients after OT are limited. In line with previous reports [45,46], the majority of patients in the present study were completely satisfied with their final smile aesthetics. Interestingly, patients scored their own smile higher than periodontists did. This suggests that clinicians are more critical in their aesthetic perceptions than patients in general. Moreover, the high level of patient satisfaction enforces the negative influence of periodontitis on their quality of life in terms of psychological discomfort and functional limitation [2,47]. A systematic review reported that the emotional and psychological aspects were those that benefited most from periodontal treatment [48].

## 5. Conclusions

Within the limitations of this retrospective study, the following conclusions can be drawn:

- PD and CAL obtained with the active periodontal treatment remained stable after OT and during the 8.9 years of follow-up;
- all anterior teeth with PTM were successfully retained during OT and SPC;
- intruded teeth experienced stability in MBL with some degree of root resorption;
- patients were highly satisfied with their own final smile aesthetics;
- the perception of aesthetics differed between patients and clinicians.

In conclusion, OT plays a central role in the multidisciplinary management of Stage IV periodontitis to re-establish a healthy and functional dentition, but it is a demanding procedure requiring specialized skills. While the reduced anatomical support of the periodontally involved teeth does not represent a limit for OT, it is imperative to move teeth only if the periodontal health has been re-established and if patients can demonstrate an adequate level of oral hygiene. Close monitoring of periodontal conditions is paramount during active OT and during the retention phase to avoid any detrimental effect of tooth movement on the periodontium, which may occur if inflammation is present.

## Figures and Tables

**Table 1:** Full-mouth periodontal clinical variables (mean ± SD) over the observational period.

Variables	T0 Baseline	T1 Active Periodontal Treatment	ΔT0–T1	T2 Orthodontic Treatment	ΔT0–T2	T3 Last Follow-Up	ΔT0–T3
FMPS (%)	53.1 ± 17.9 *	15.9 ± 2.9	37.1 ± 17.7 ^†^	16.9 ± 2.7	36.2 ± 17.9 ^†^	18.2 ± 4.3	34.9 ± 18.4 ^†^
FMBS (%)	34.2 ± 16.7 *	8.7 ± 3.6	25.5 ± 14.8 ^†^	8.4 ± 3.1	25.8 ± 15.1 ^†^	7.5 ± 3.2	26.7 ± 15.5 ^†^
PD (mm)	4.2 ± 0.9 *	3.1 ± 0.3	1.1 ± 0.8 ^†^	2.9 ± 0.4	1.3 ± 0.9 ^†^	3.0 ± 0.3	1.2 ± 0.8 ^†^
CAL (mm)	5.0 ± 1.1 *	4.4 ± 0.7	0.6 ± 0.9 ^‡^	4.1 ± 0.8	0.8 ± 1.1 ^†^	4.4 ± 0.9	0.6 ± 1.0 ^‡^
REC (mm)	0.8 ± 0.6 *	1.3 ± 0.6	−0.5 ± 0.5 ^†^	1.2 ± 0.6	−0.4 ± 0.6 ^†^	1.4 ± 0.7	−0.6 ± 0.7 ^†^
N° teeth	21.9 ± 4.6 *	19.5 ± 5.0	2.4 ± 2.2 ^†^	19.3 ± 4.9	2.6 ± 2.2 ^†^	19.1 ± 4.8	2.8 ± 2.1 ^†^

FMPS = full-mouth plaque score; FMBS = full-mouth bleeding score; PD = probing depth; CAL = clinical attachment level; REC = gingival recession; SD = standard deviation; * *p* < 0.005, *p* values represent changes among the four time points; ^†^  *p* ≤ 0.001, *p* values represent longitudinal changes from T_0_; **^‡^**  *p* ≤ 0.05, *p* values represent longitudinal changes from T_0_.

**Table 2:** Periodontal clinical and radiographic changes (mean ± SD) at anterior teeth with PTM.

Variables	T0 Baseline	T1 Active Periodontal Treatment	ΔT0–T1	T2 Orthodontic Treatment	ΔT0–T2	T3 Last Follow-Up	ΔT0–T3
PD (mm)	4.3 ± 1.0 *	2.9 ± 0.5	1.4 ± 0.9 ^‡^	2.9 ± 0.6	1.4 ± 1.0 ^‡^	2.8 ± 0.5	1.5 ± 0.8 ^‡^
CAL (mm)	5.1 ± 1.4 *	4.3 ± 1.0	0.8 ± 0.9 ^‡^	4.3 ± 1.1	0.8 ± 1.0 ^‡^	4.4 ± 1.1	0.7 ± 1.0
REC (mm)	0.8 ± 0.7 *	1.4 ± 0.8	−0.6 ± 0.5 ^‡^	1.4 ± 0.8	−0.6 ± 0.7 ^‡^	1.6 ± 0.9	−0.8 ± 0.7 ^‡^
CEJ-RA (mm)	16.3 ± 1.6 *	—	—	15.2 ± 1.9	1.1 ± 0.8 ^‡^	14.7 ± 1.8	1.6 ± 1.2 ^‡^
Residual bone (%)	65.2 ± 10.9 ^†^	—	—	64.6 ± 9.4	−0.6 ± 6.9	65.1 ± 10.3	−0.1 ± 7.7

PD = probing depth; CAL = clinical attachment level; REC = gingival recession; CEJRA = root length; SD = standard deviation; * *p* < 0.001, *p* values represent changes among the time points; ^†^  *p* > 0.05, *p* values represent changes among the three time points; ^‡^  *p* ≤ 0.01, *p* values represent longitudinal changes from T0.

**Table 3:** Number of extracted teeth and reasons for tooth extraction according to the treatment phase (N, (%)).

Reason for Extraction	T0 to T1	T1 to T2	T2 to T3	Total
Periodontal lesion	51 (71%)	4 (100)	0	55 (67%)
Root fracture	2 (3%)	0	6 (100)	8 (10%)
Endodontic failure	13 (18%)	0	0	13 (16%)
Non-restorable carious lesion	6 (8%)	0	0	6 (7%)

T0 = baseline; T1 = re-evaluation after active periodontal treatment; T2 = re-evaluation after orthodontic treatment; T3 = final follow-up.

## Data Availability

The data presented in this study are available on request from the corresponding author.

## References

[1] Kassebaum N.J., Bernabé E., Dahiya M., Bhandari B., Murray C.J., Marcenes W. (2014). Global burden of severe periodontitis in 1990-2010: A systematic review and meta-regression. J. Dent. Res..

[2] Tonetti M.S., Jepsen S., Jin L., Otomo-Corgel J. (2017). Impact of the global burden of periodontal diseases on health, nutrition and wellbeing of mankind: A call for global action. J. Clin. Periodontol..

[3] Meyle J., Chapple I. (2015). Molecular aspects of the pathogenesis of periodontitis. Periodontol. 2000.

[4] Papapanou P.N., Sanz M., Buduneli N., Dietrich T., Feres M., Fine D.H., Flemmig T.F., Garcia R., Giannobile W.V., Graziani F. (2018). Periodontitis: Consensus report of workgroup 2 of the 2017 World Workshop on the classification of periodontal and peri-implant diseases and conditions. J. Clin. Periodontol..

[5] Tonetti M.S., Greenwell H., Kornman K.S. (2018). Staging and grading of periodontitis: Framework and proposal of a new classification and case definition. J. Clin. Periodontol..

[6] Brunsvold M.A. (2005). Pathologic tooth migration. J. Periodontol..

[7] Towfighi P.P., Brunsvold M.A., Storey A.T., Arnold R.M., Willman D.E., McMahan C.A. (1997). Pathologic migration of anterior teeth in patients with moderate to severe periodontitis. J. Periodontol..

[8] Gkantidis N., Christou P., Topouzelis N. (2010). The orthodontic periodontic interrelationship in integrated treatment challenges: A systematic review. J. Oral Rehabil..

[9] Sanz M., Herrera D., Kebschull M., Chapple I., Jepsen S., Berglundh T., Sculean A., Tonetti M.S. (2020). EFP Workshop Participants and Methodological Consultants. Treatment of stage I-III periodontitis-the EFP S3 level clinical practice guideline. J. Clin. Periodontol..

[10] Herrera D., Sanz M., Kebschull M., Jepsen S., Sculean A., Berglundh T., Papapanou P.N., Chapple I., Tonetti M.S. (2022). EFP Workshop Participants and Methodological Consultant. Treatment of stage IV periodontitis: The EFP S3 level clinical practice guideline. J. Clin. Periodontol..

[11] Martin C., Celis B., Ambrosio N., Bollain J., Antonoglou G.N., Figuero E. (2022). Effect of orthodontic therapy in periodontitis and non-periodontitis patients: A systematic review with meta-analysis. J. Clin. Periodontol..

[12] Aimetti M., Garbo D., Ercoli E., Grigorie M.M., Citterio F., Romano F. (2020). Long-term prognosis of severely compromised teeth following combined periodontal and orthodontic treatment: A retrospective study. Int. J. Periodontics Restor. Dent..

[13] Papageorgiou S.N., Antonoglou G.N., Michelogiannakis D., Kakali L., Eliades T., Madianos P. (2022). Effect of periodontal–orthodontic treatment of teeth with pathological tooth flaring, drifting, and elongation in patients with severe periodontitis: A systematic review with meta-analysis. J. Clin. Periodontol..

[14] Ingber J.S. (1974). Forced Eruption: Part I. A method of treating isolated one and two wall infrabony osseous defects-rationale ands case report. J. Periodontol..

[15] Melsen B. (2001). Tissue reaction to orthodontic tooth movement—A new paradigm. Eur. J. Orthod..

[16] Kondo T., Hotokezaka H., Hamanaka R., Hashimoto M., Nakano-Tajima T., Arita K., Kurohama T., Hino A., Tominaga J.Y., Yoshida N. (2017). Types of tooth movement, bodily or tipping, do not affect the displacement of the tooth’s center of resistance but do affect the alveolar bone resorption. Angle Orthod..

[17] Kim K., Heimisdottir K., Gebauer U., Persson G.R. (2010). Clinical and microbiological findings at sites treated with orthodontic fixed appliances in adolescents. Am. J. Orthod. Dentofac. Orthop..

[18] Langford S.R. (1982). Root resorption extremes resulting from clinical RME. Am. J. Orthod..

[19] Fuhrmann R.A.W. (2002). Three-dimensional evaluation of periodontal remodelling during orthodontic treatment. Semin. Orthod..

[20] Gorbunkova A., Pagni G., Brizhak A., Farronato G., Rasperini G. (2016). Impact of orthodontic treatment on periodontal tissues: A narrative review of multidisciplinary literature. Int. J. Dent..

[21] Zasciurinskiene E., Lindsten R., Slotte C., Bjerklin K. (2016). Orthodontic treatment in periodontitis-susceptible subjects: A systematic literature review. Clin. Exp. Dent. Res..

[22] Chen M.X., Zhong Y.J., Dong Q.Q., Wong H.M., Wen Y.F. (2021). Global, regional, and national burden of severe periodontitis, 1990-2019: An analysis of the global burden of disease study 2019. J. Clin. Periodontol..

[23] Hirschfeld J., Reichardt E., Sharma P., Hilber A., Meyer-Marcotty P., Stellzig-Eisenhauer A., Schlagenhauf U., Sickel F. (2019). Interest in orthodontic tooth alignment in adult patients affected by periodontitis: A questionnaire-based cross-sectional pilot study. J. Periodontol..

[24] Burstone C.J., Koenig H.A. (1976). Optimizing anterior and canine retraction. Am. J. Orthod..

[25] Minch L.E., Sarul M., Nowak R., Kawala B., Antoszewska-Smith J. (2017). Orthodontic intrusion of periodontally-compromised maxillary incisors: 3-dimensional finite element method analysis. Adv. Clin. Exp. Med..

[26] Shroff B., Yoon W.M., Lindauer S.J., Burstone C.J. (1997). Simultaneous intrusion and retraction using a three-piece base arch. Angle Orthod..

[27] Björn H., Halling A., Thyberg H. (1969). Radiographic assessment of marginal bone loss. Odontol. Revy.

[28] Kokich V.C., Spear F.M. (1997). Guidelines for managing the orthodontic-restorative patient. Semin. Orthod..

[29] Ericsson I., Thilander B., Lindhe J., Okamoto H. (1977). The effect of orthodontic tilting movements on the periodontal tissues of infected and non-infected dentitions in dogs. J. Clin. Periodontol..

[30] Polson A., Caton J., Polson A.P., Nyman S., Novak J., Reed B. (1984). Periodontal response after tooth movement into intra-bony defects. J. Periodontol..

[31] Mathews D.P., Kokich V.G. (1997). Managing treatment for the orthodontic patient with periodontal problems. Semin. Orthod..

[32] Pini Prato G.P., Chambrone L. (2020). Orthodontic treatment in periodontal patients: The use of periodontal gold standards to overcome the “grey zone”. J. Periodontol..

[33] Boyd R.L., Murray P., Robertson P.B. (1989). Effect of rotary electric toothbrush versus manual toothbrush on periodontal status during orthodontic treatment. Am. J. Orthod. Dentofac. Orthoped..

[34] Boyd R.L., Leggott P.J., Quinn R.S., Eakle W.S., Chambers D. (1989). Periodontal implications of orthodontic treatment in adults with reduced or normal periodontal tissues versus those of adolescents. Am. J. Orthod. Dentofac. Orthop..

[35] Melsen B., Agerbaek N., Markenstam G. (1989). Intrusion of incisors in adult patients with marginal bone loss. Am. J. Orthod. Dentofacial. Orthop..

[36] Corrente G., Abundo R., Re S., Cardaropoli D., Cardaropoli G. (2003). Orthodontic movement into infrabony defects in patients with advanced periodontal disease: A clinical and radiological study. J. Periodontol..

[37] Re S., Corrente G., Abundo R., Cardaropoli D. (2000). Orthodontic treatment in periodontally compromised patients: 12-year report. Int. J. Periodontics Restor. Dent..

[38] Melsen B., Agerbaek N., Eriksen J., Terp S. (1988). New attachment through periodontal treatment and orthodontic intrusion. Am. J. Orthod. Dentofac. Orthop..

[39] Papageorgiou S.N., Koletsi D., Illiadi A., Peltomaki T., Eliades T. (2020). Treatment outcome with orthodontic aligners and fixed appliances: A systematic review with meta analysis. Eur. J. Orthod..

[40] Kloukos D., Roccuzzo A., Stähli A., Sculean A., Katsaros C., Salvi G.E. (2022). Effect of combined periodontal and orthodontic treatment of tilted molars and of teeth with intra-bony and furcation defects in stage-IV periodontitis patients: A systematic review. J. Clin. Periodontol..

[41] Zasčiurinskiene E., Basevičiene N., Lindsten R., Slotte C., Jansson H., Bjerklin K. (2018). Orthodontic treatment simultaneous to or after periodontal cause-related treatment in periodontitis susceptible patients. Part I: Clinical outcome. A randomized clinical trial. J. Clin. Periodontol..

[42] Aimetti M., Garbo D., Vidotto C., Bongiovanni L., Citterio F., Mariani G.M., Baima G., Romano F. (2022). Combined periodontal and orthodontic treatment of severely compromised teeth in Stage IV periodontitis patients: How far can we go?. Int. J. Periodontics Restor. Dent..

[43] Melsen B. (1986). Tissue reaction following application of extrusive and intrusive forces to teeth in adult monkeys. Am. J. Orthod..

[44] Artun J., Urbye K.S. (1988). The effect of orthodontic treatment on periodontal bone support in patients with advanced loss of marginal periodontium. Am. J. Orthod. Dentofac. Orthop..

[45] Riedmann T., Georg T., Berg R. (1999). Adult patients′ view of orthodontic treatment outcome compared to professional assessments. J. Orofac. Orthop..

[46] Lee R., Hwang S., Lim H., Cha J.Y., Kim K.H., Chung C.J. (2018). Treatment satisfaction and its influencing factors among adult orthodontic patients. Am. J. Orthod. Dentofac. Orthop..

[47] Ferreira M.C., Dias-Pereira A.C., Branco-de-Almeida L.S., Martins C.C., Paiva S.M. (2017). Impact of periodontal disease on quality of life: A systematic review. J. Periodontal. Res..

[48] Shanbhag S., Dahiya M., Croucher R. (2012). The impact of periodontal therapy on oral health-related quality of life in adults: A systematic review. J. Clin. Periodontol..

